# Supplementary material for: Synthesis and Validation of a Bioinspired Catechol-Functionalized Pt(IV) Prodrug for Preclinical Intranasal Glioblastoma Treatment
Source: Cancers (Basel). 2022 Jan 14;14(2):410. doi: 10.3390/cancers14020410 (PMC8774041; doi:10.3390/cancers14020410)
Supplement: Supplementary file 1 [file cancers-14-00410-s001.zip › cancers-1503077-supplementary.pdf]

Supplementary Materials

Synthesis and Validation of a Bioinspired Catechol - Functionalized Pt(IV) Prodrug for Preclinical Intranasal Glioblastoma Treatment

Xiaoman Mao, Shuang Wu, Pilar Calero-Pérez, Ana P. Candiota, Paula Alfonso, Jordi Bruna, Victor J. Yuste, Julia Lorenzo, Fernando Novio and Daniel Ruiz-Molina

1. Characterization Data of Complex 1

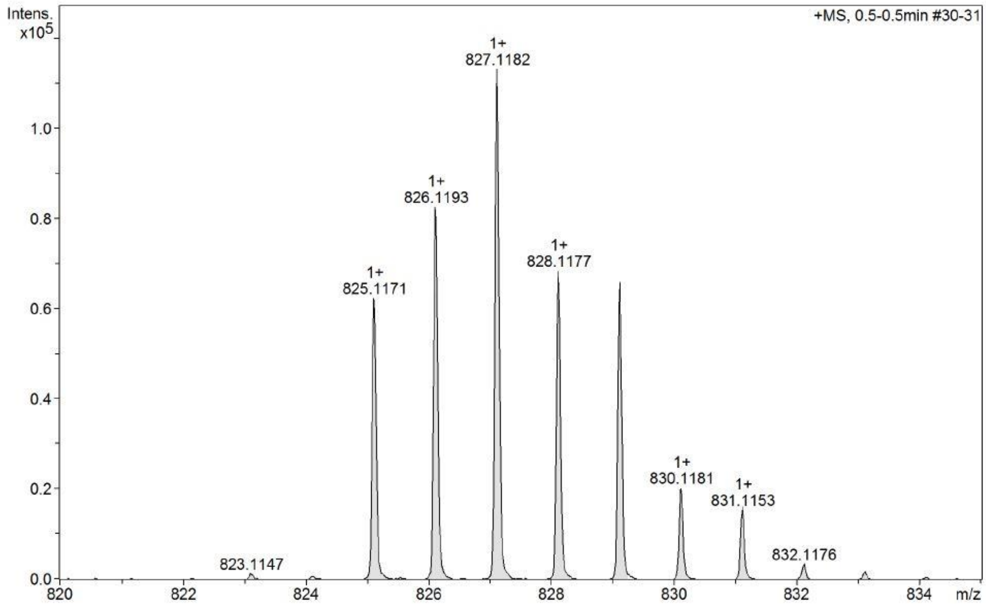

Figure S1. High-resolution electrospray ionization mass spectrometry (HR-ESI-MS) spectrum of complex **1**; main peak corresponds to $[C_{24}O_{10}H_{34}N_4Cl_2PtNa\text{-}H]^+$ calculated 827.1276.

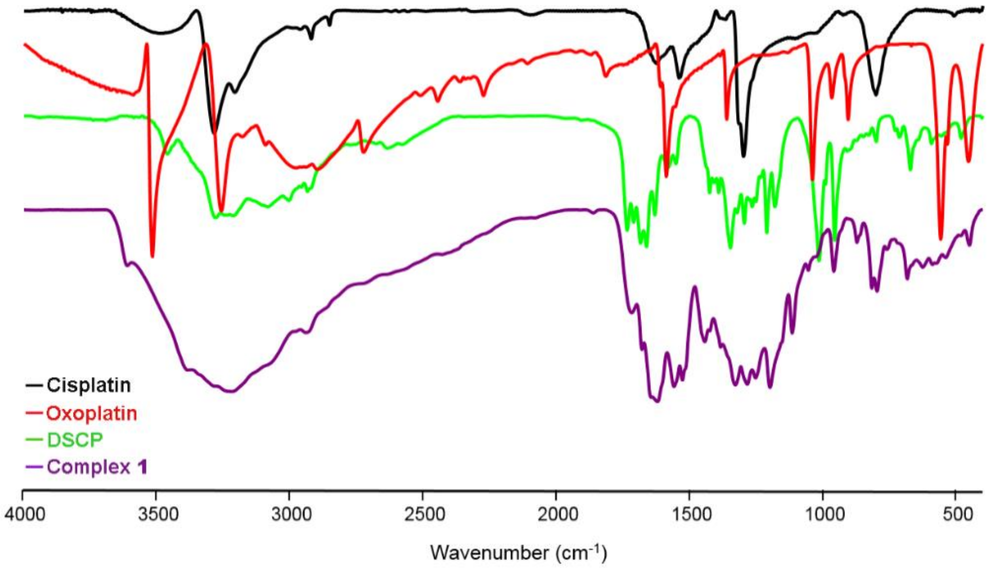

Figure S2. FT-IR spectra of cisplatin, oxoplatin, DSCP and complex **1**.

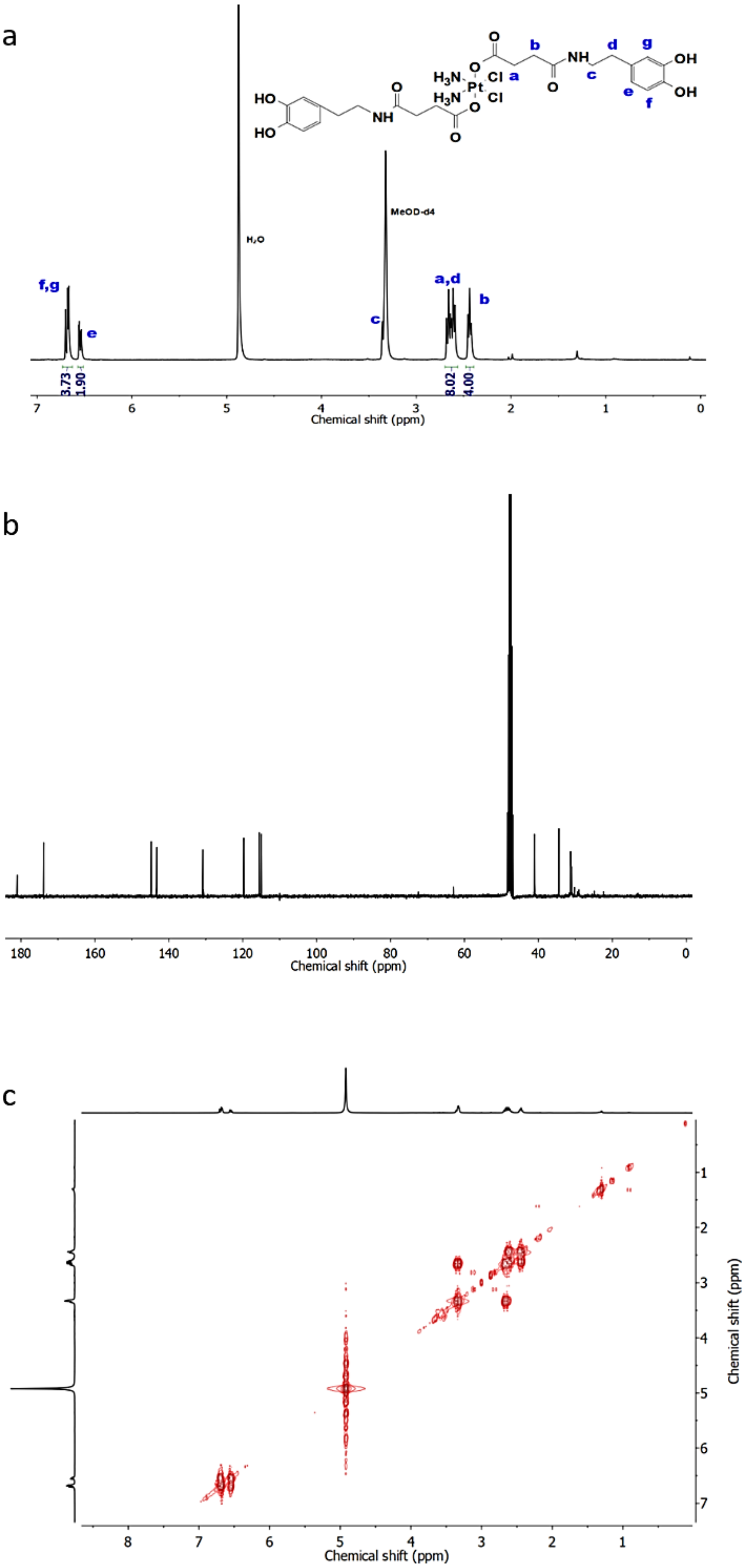

Figure S3. (**a**) ^{1}H, (**b**) ^{13}C, and (**c**) 2D H-1H COSY (COrrelated SpectroscopY) NMR spectra of complex **1**.

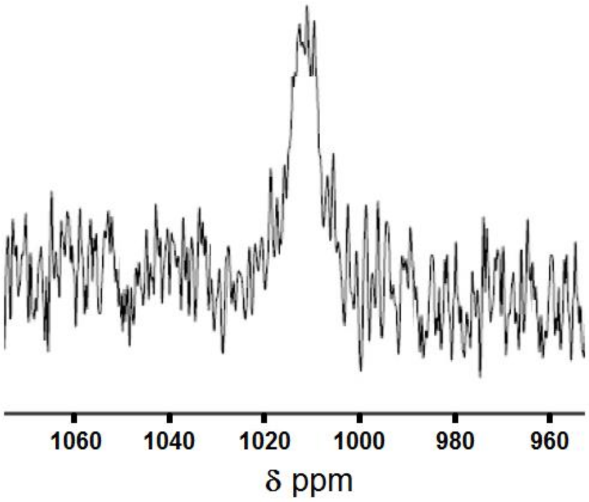

Figure S4. ^{195}Pt NMR spectrum of complex **1**.

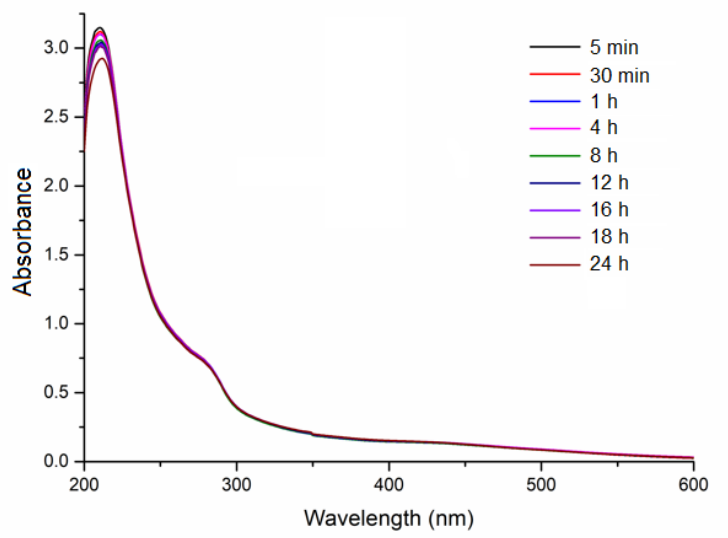

Figure S5. UV-Vis spectra of complex **1** in phosphate-buffered saline solution (PBS, pH 7.4) at 37 °C recorded at different time points.

2. In Vitro Studies

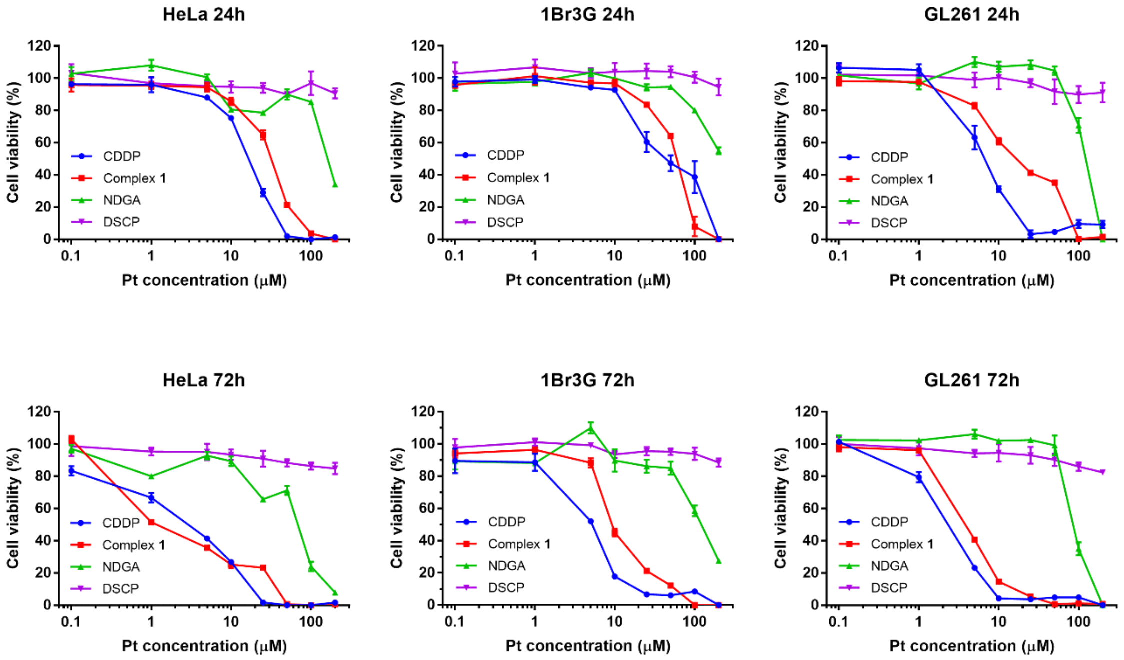

Figure S6. Cytotoxicity of complex **1**, cisplatin (CDDP), nordihydroguaiaretic acid (NDGA) and disuccinatocisplatin (DSCP) against a panel of cell lines for 24 h and 72 h. All data represented mean ± SE of three independent experiments.

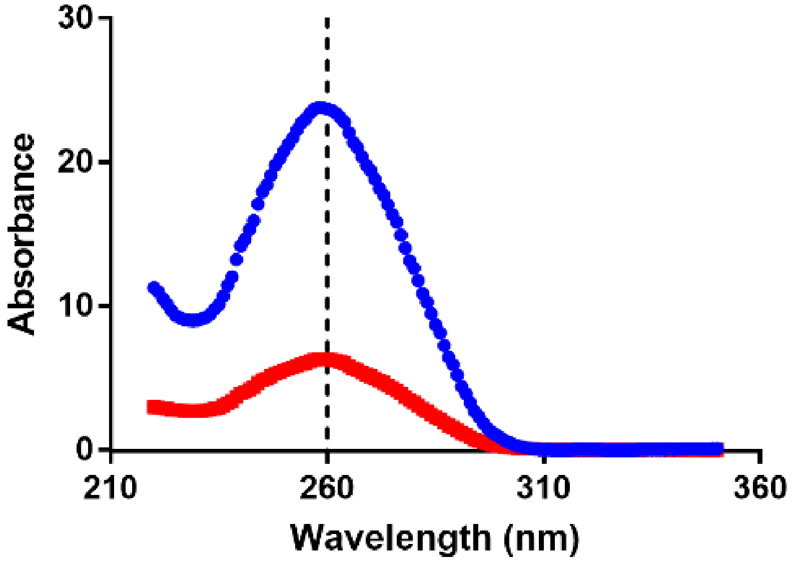

Figure S7. Representative absorbance spectra of DNA concentrations determined by NanoDrop 1000 UV-vis spectrometer. Blue curve: 1184.7 ng/μL, red curve: 312.75 ng/μL.

3. In Vivo Tolerability Studies

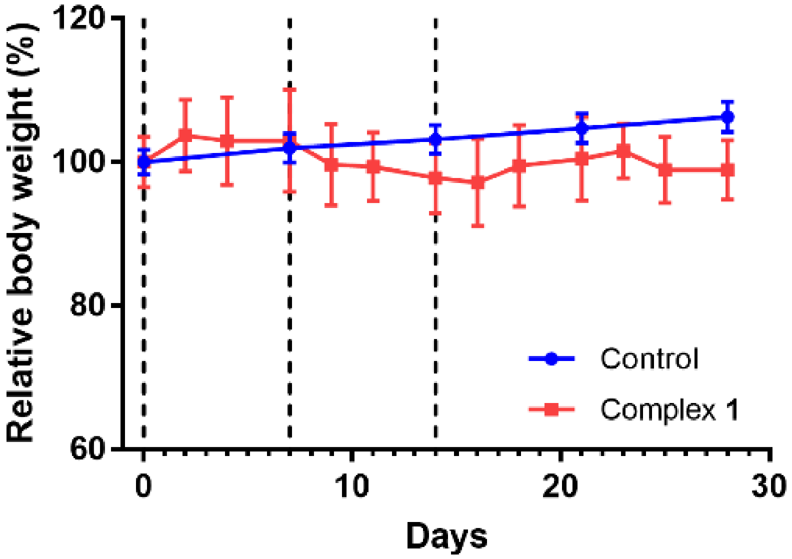

Figure S8. Tolerability assessment of mice for complex **1** over four weeks. Three escalated doses (0.9, 1.2 and 1.5 mg/kg) were given once a week via intranasal route indicated by dash line, *n* = 3 for complex **1** group. For control group, *n* = 40, data adapted from Jackson Laboratory (https://www.jax.org/jax-mice-and-services/strain-data-sheet-pages/body-weight-chart-000664, accessed on 24 November 2021). Data represented as mean ± SE. Dash lines indicated days for administration. Dashed lines indicate administration points.

4. Therapeutic Protocols and Acquisition Schemes

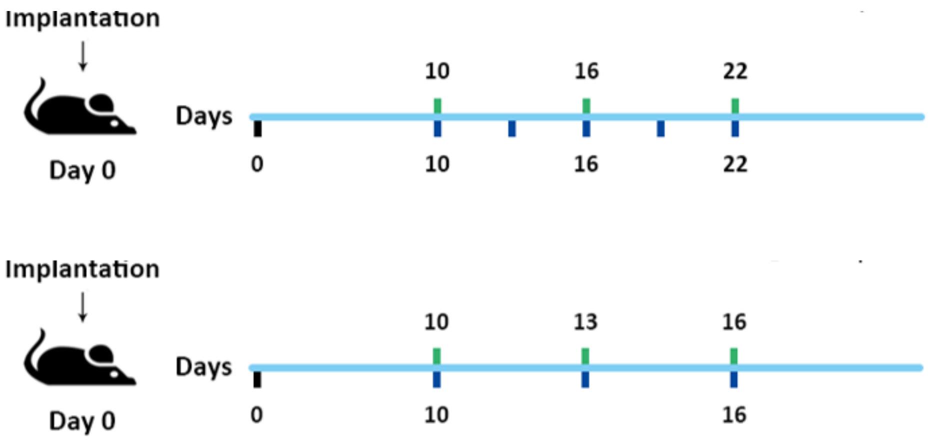

Figure S9. (**Top**) Immune-enhancing metronomic schedule (IMS) used for GL261 glioblastoma (GB) therapy in mice. Complex **1** was administered every 6 days (green labels) while tumor volumes acquired by high-resolution magnetic resonance imaging (MRI) $T2_W$ were recorded twice per week (blue labels). (**Bottom**) Adjusted intensive treatment schedule for GL261 GB therapy in mice. Complex **1** was administered intranasally now every 3 days, same interval used to measure tumor volumes by high-resolution MRI $T2_W$.

5. Tumor Volume Monitoring by High-Resolution $T2_w$ MRI

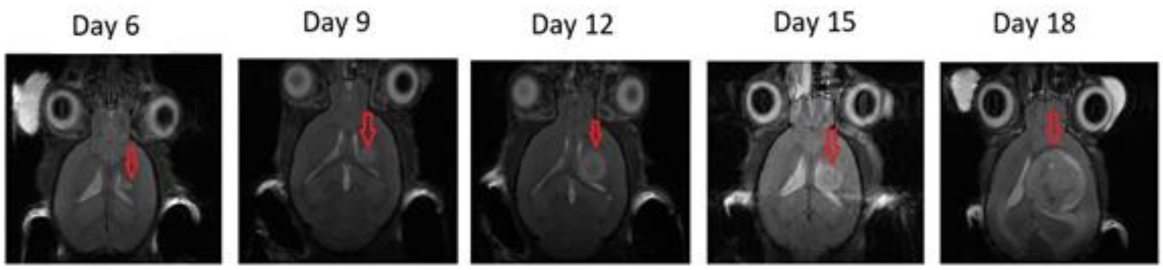

Figure S10. Representative evolution of tumor volume (red arrow) recorded by high-resolution $T2_w$ MRI acquisitions at different days for a GL261 GB bearing mouse.

© 2021 by the authors. Licensee MDPI, Basel, Switzerland. This article is an open access article distributed under the terms and conditions of the Creative Commons Attribution (CC BY) license (http://creativecommons.org/licenses/by/4.0/).
